# The Advanced Confidentiality Engine as a Scalable Tool for the Pseudonymization of Biomedical Data in Translational Settings: Development and Usability Study

**DOI:** 10.2196/71822

**Published:** 2025-11-05

**Authors:** Armin Müller, Eric Wündisch, Felix Nikolaus Wirth, Sophie Meier zu Ummeln, Joachim Weber, Fabian Prasser

**Affiliations:** 1 Medical Informatics Group Berlin Institute of Health at Charité - Universitätsmedizin Berlin Berlin Germany; 2 Core Unit Treuhandstelle Berlin Institute of Health at Charité - Universitätsmedizin Berlin Berlin Germany; 3 Center for Stroke Research Berlin Charité - Universitätsmedizin Berlin Berlin Germany; 4 Department of Neurology Charité - Universitätsmedizin Berlin Berlin Germany; 5 Partner Site Berlin German Centre for Cardiovascular Research Berlin Germany

**Keywords:** health information management, information storage, information retrieval, biomedical research, data privacy, pseudonymization

## Abstract

**Background:**

Pseudonymization refers to a process in which data that directly identify individuals, such as names and addresses, are stored separately from data needed for scientific purposes. The connection between both types of data is maintained through a protected link, represented by pseudonyms. This is a central data protection method in translational research, which enables researchers to collect, process, and share data while adhering to “data protection by design and by default” and data minimization best practices. However, integrating pseudonymization into high-throughput data processing workflows is challenging, and open-source solutions are rare. A typical example is the need to pseudonymize millions of electronic health records for secondary use in translational research platforms.

**Objective:**

This paper introduces the Advanced Confidentiality Engine (ACE), a highly scalable open-source pseudonymization service focused on creating and managing the protected link between identifying and research data.

**Methods:**

ACE has been designed to have a lean architecture, consisting of a compact database schema that mimics the design of data warehouses. It is implemented using modern open-source software technologies and provides a Representational State Transfer application programming interface. Among its features are a fine-grained access control mechanism, a domain-based structuring of pseudonyms with attribute inheritance, and a comprehensive audit trail. We performed a structured evaluation to study ACE’s scalability under various workload scenarios.

**Results:**

For generating protected links, ACE supports 9 different pseudonymization algorithms, including approaches based on cryptographic primitives and random number generation. Pseudonyms can be encoded using different alphabets that can be combined with check digits. Pseudonyms can be annotated with metadata, such as validity periods, and those properties can be inherited through a hierarchical domain structure. As all information is persisted by ACE, it supports pseudonymization and depseudonymization, for which access can be controlled individually. Our experiments show that ACE is able to handle around 6000 transactions per second in different workload settings. ACE combines the efficiency of cryptography-based pseudonymization methods with the flexibility of persistence-based approaches.

**Conclusions:**

ACE is a modern and highly scalable implementation of a pseudonymization service tailored toward the specific requirements in biomedical research. It is available as open-source software. As the space of openly available pseudonymization services is limited, we believe that ACE is valuable to institutions establishing or improving their translational data infrastructure.

## Introduction

### Background

Biomedical research relies on the effective collection, management, sharing, and analysis of large datasets [1-4]. The importance of pseudonymization in infrastructures for reusing health data in research is demonstrated by its role in several large-scale data platforms, for example, at the Vanderbilt University Medical Center in the United States [5], the Medical Informatics Initiative (MII) in Germany (see Prasser et al [6] for an example from the Data Integration for Future Medicine consortium in the MII), and the Secure Anonymised Information Linkage Databank in the United Kingdom [7]. At the same time, biomedical and health datasets are often highly sensitive and require protection against disclosure and privacy threats [8,9]. As a result, various laws and best practices suggest to implement pseudonymization as a data protection mechanism when collecting, sharing, and processing biomedical data, hence following “data protection by design and by default” as well as data minimization principles [10,11], which can be further combined with anonymization during later steps, for example, when data are shared [12].

**Figure 1:**
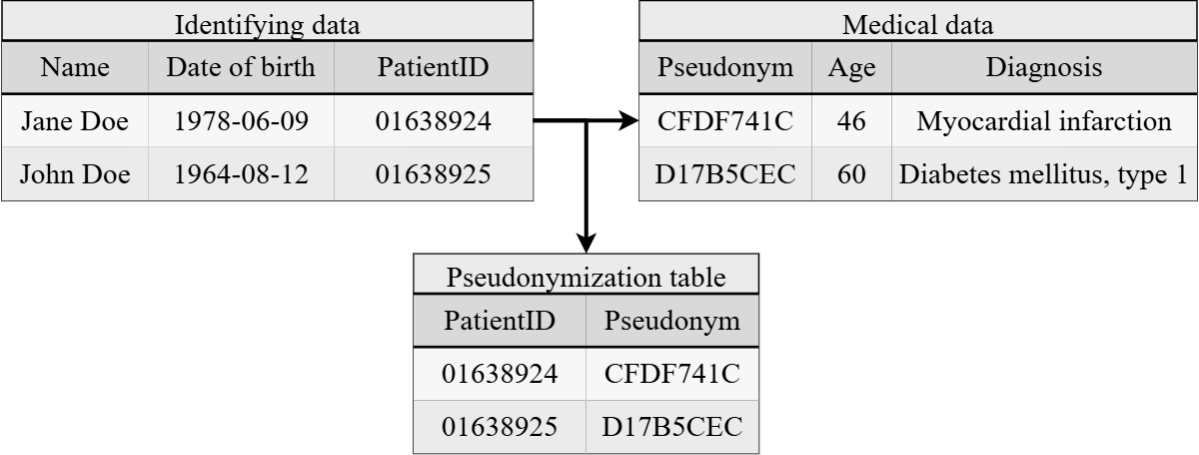
Pseudonymization example. Medical data are separated from identifying data, and a protected link is introduced. This protected link is stored securely using a mapping table, which ensures that depseudonymization is possible when needed.

Pseudonymization refers to a process in which data that directly identify individuals, such as names and addresses, are separated from data needed for scientific analyses, and a protected link is introduced. This link is established using secure identifiers, known as pseudonyms [13]. These pseudonyms are stored securely, enabling authorized personnel to reverse the pseudonymization when necessary, such as for recontacting patients in the case of incidental findings. Creating and managing these links at scale require customizable tools that support different application scenarios (see Performance Evaluation section). A simple example is illustrated in Figure 1, where medical data are separated from identifying data, and a protected link is introduced, which is stored separately in a pseudonymization table.

In medical research, pseudonymization is often implemented by integrating data processing workflows with dedicated stand-alone services. As detailed in a recent review by Abu Attieh et al [14], a variety of such services has been described in the literature. However, as our comparison in the Comparison With Prior Work section shows, these are not suited to support the scenario addressed in this paper, that is, the high-throughput, on-premise pseudonymization of large-scale datasets in a flexible manner. On one hand, services like the generic Pseudonym Administration Service (gPAS) [15] and Mainzelliste [16] provide core pseudonymization functionalities but are architected for study-specific deployments and do not easily scale to the size of institution-wide data repositories. On the other hand, scalable solutions such as Secure Privacy-Preserving Identity Management in Distributed Environments for Research (SPIDER) [17] are often only provided as software-as-a-service offerings, preventing local deployment. Moreover, with the exception of the ORCHESTRA Pseudonymization Tool (OPT) [18], which targets manual use in individual studies rather than automated, large-scale processing, performance benchmarks are rarely reported. Finally, support for advanced features needed in diverse research scenarios, such as bulk processing, management of metadata in multiple pseudonym spaces, or configurable pseudonym properties, is inconsistent across existing solutions.

An alternative approach for high-throughput pseudonymization is the use of cryptographic operations directly within the underlying data processing platforms, as this allows pseudonyms to be derived quickly from identifying data. One example is hashing algorithms, for example, implemented to link electronic health record data and biosamples at scale at the Vanderbilt University Medical Center [19]. However, hashing is a 1-way approach that prevents depseudonymization. As an alternative, encryption methods can be used. However, with hashing and encryption methods, it becomes challenging to maintain oversight over who resolves which pseudonym in which context and to invalidate protected links, for example, after a certain time period. Moreover, hashes and cryptographic tokens are hard to use in some contexts, for example, if there is a need to transmit them via phone or enter them into case report forms.

### Objective

In this paper, we introduce the Advanced Confidentiality Engine (ACE), a highly scalable open-source pseudonymization service for health data, which offers functionalities for creating and managing pseudonyms and securely storing the link between identifying and research data. ACE has been designed to combine the best of both worlds, that is, hashing or cryptographic-, and persistence-based approaches. It provides high performance together with access control, monitoring, and auditing as well as support for a wide range of pseudonymization algorithms and structures through a modern Representational State Transfer (REST) interface. We describe the basic design of ACE and evaluate its performance in different workload scenarios.

## Methods

To ensure that our solution properly addresses real-world challenges, we adopted a requirement-driven design followed by an iterative implementation process. We derived these requirements from our experiences running a large translational research data platform.

### Fundamental Requirements

From our experiences in supporting clinical studies [20] as well as health data lakes [6], we derived three sets of fundamental requirements:

1. Performance and scalability: The service needs to be able to handle common workloads in clinical studies with short response times and low resource use. At the same time, the service should also be able to scale to big data workloads, enabling thousands of pseudonyms to be generated, resolved, or modified per second.
2. Customizability and flexibility: Usually, several pseudonyms are required to protect different types of data with different identifiers, for example, laboratory reports and imaging studies, for the same patient or participant. Hence, the service needs to support complex pseudonym setups as well as simple mapping tables. Moreover, it must be configurable to support short and human-readable pseudonyms of different lengths with and without checksums as well as common hashing algorithms.
3. Control and compliance: The service must provide functionalities to control who can create, update, read, or delete which pseudonyms. Furthermore, it must be possible to invalidate links between identifiers and pseudonyms, for example, using a validity period. All read and write accesses to pseudonyms must be logged in an audit trail.

On the nonfunctional side, we aimed for a software that can be distributed under a permissive open-source license and is based on modern software technologies and components.

### Domain Model

**Figure 2:**
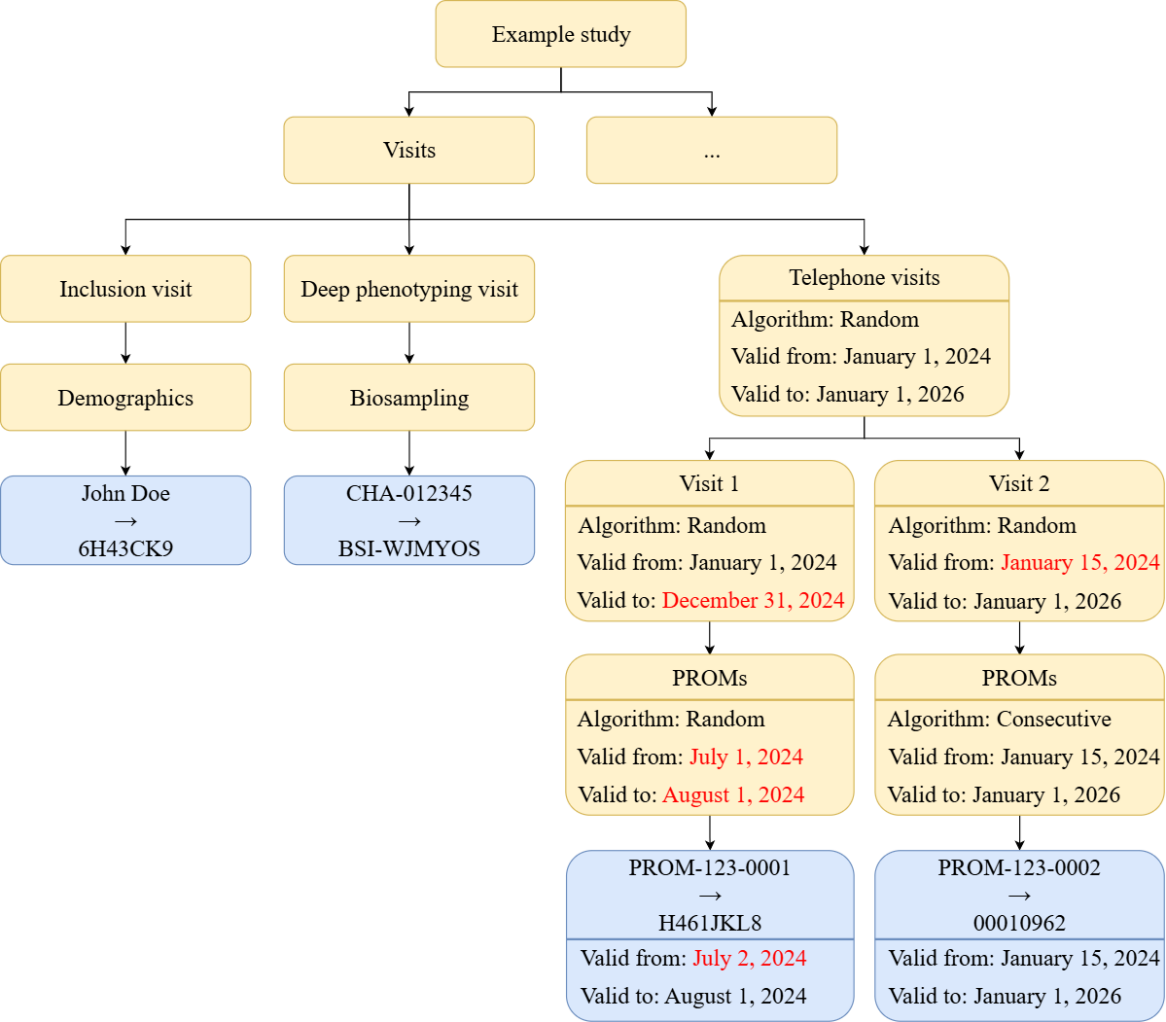
Domains (represented in yellow) and pseudonym objects (in blue) in ACE. This example shows an instantiation of the model in ACE. The visits subtree shows an example of attribute inheritance. Here, values written in black are inherited from the parent domain, and values written in red have been overwritten by manually provided values. ACE: Advanced Confidentiality Engine; PROM: patient-reported outcome measure.

Clinical studies and also routine health care data can often be categorized into different contexts, depending on the time of collection, the source of data, or the data modality. For example, prospective studies usually collect different types of data and biosamples during different study visits. In health care, for instance, laboratory reports, imaging studies, and monitoring signals are important types of data that often use different identifiers. To account for this, we adopted the concept of “domains” in ACE, which is also used in other pseudonymization services [15]. Domains can be nested to, for example, represent that different types of data have been collected at different points in time but within the context of the same study. In ACE, domains are also holding large parts of the configuration options. For example, different domains can be configured to use different pseudonymization algorithms and have different validity periods. An example is provided in Figure 2.

The example illustrates a (simplified) study, which is organized into different visits. Basic patient demographics of the study participants are collected in the inclusion visit. The participant’s name is pseudonymized into a prefix-less 8-character pseudonym. The participants included in the study then take part in a deep phenotyping procedure. The identifiers for the collected biosamples in this visit are pseudonymized and stored in the “Biosampling” domain. Further data are then collected via telephone visits, where, as an example, patient-reported outcome measures (PROMs) are collected. In the figure, we illustrate some configuration options for these domains. As can be seen, properties of domains are inherited to subdomains and pseudonyms in subdomains but can also be overwritten (indicated in red; see Attribute Inheritance section). The resulting pseudonyms are a random string (“H461JKL8”) and a counter padded to 8 characters (“00010962”). Domains that semantically cluster information are shown in yellow. Individual pseudonym mappings are shown in blue.

### Attribute Inheritance

Generated pseudonyms are always attributed to a single domain, which, as mentioned, stores all associated configuration options. In practice, subdomains often share properties with their parent domains, for example, validity periods. Therefore, domain and pseudonym properties in ACE support inheritance. This means that when a new domain or pseudonym is created, it can be specified to inherit configuration options from its parent domain. Values are not only inherited once, but changes will propagate to child domains with inherited values as well if ACE is configured to do so. If domain-specific or pseudonym-specific options are needed, inherited values can be overwritten. Updates of domain-specific properties are propagated to inheriting domains and pseudonyms when ACE is instructed to do so.

The visits subtree in Figure 2 shows an example of the attribute inheritance functionality in ACE. Here, for example, the algorithm initially set in the “telephone visits” domain is inherited to both child domains, “visit 1” and “visit 2,” and then overwritten in the child domain of “visit 2,” so that the pseudonyms generated in it are now consecutive numbers instead of strings consisting of random characters. In “visit 1,” the end date of its validity period is changed, while in “visit 2,” the validity period’s start date is changed. The change in the “visit 2” domain is then inherited to its child domain “PROMs,” which is used to store data on PROMs, and further to the pseudonym in this child domain. In the child domain of “visit 1,” on the other hand, both the start and the end date of the validity period are overwritten. Finally, the pseudonym in this domain inherited the validity period’s end date but not its start date, reflecting, for example, its creation time.

This design decision helps to ensure consistency between pseudonyms from different domains and also reduces the complexity when using ACE, as new domains and pseudonyms can be created and configured with very few parameters. At the same time, inherited configuration options and validity periods can be overwritten when necessary, for example, to invalidate a pseudonym in case of consent revocation.

### Design and Technical Implementation

**Figure 3:**
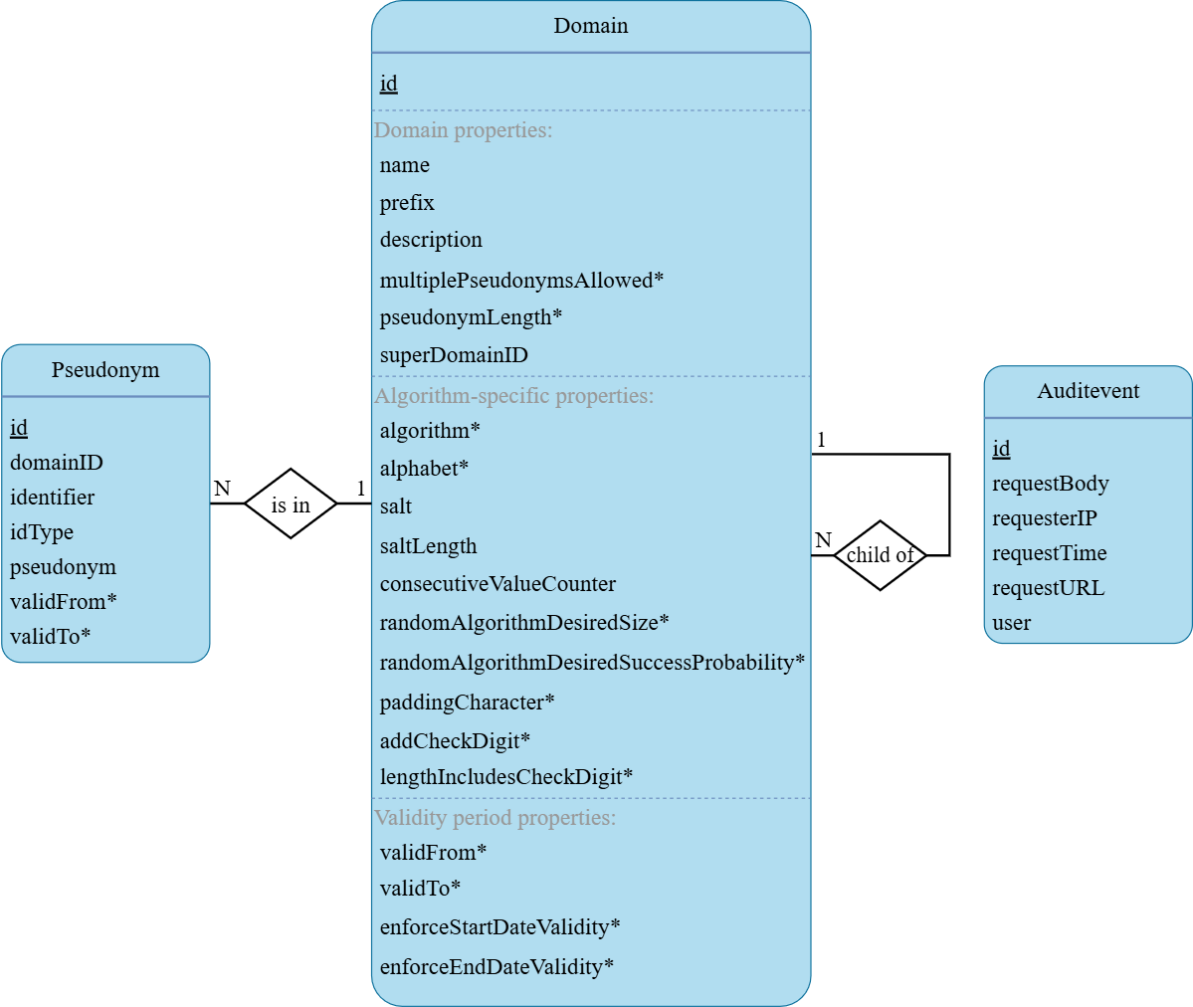
Entity-relationship diagram of the core database design of the Advanced Confidentiality Engine. An asterisk (*) next to an attribute’s name indicates that for this attribute, an additional flag is stored in the table that determines whether or not the respective attribute was inherited.

The core idea behind ACE is to combine a wide range of pseudonym generation methods, configured through domain hierarchies, with compact and highly scalable persistence of the links between input identifiers and output pseudonyms. At its core, the database schema resembles a star schema, as commonly used in data warehousing [21]. A central pseudonym table is connected to a domain table, acting as dimensions further describing the pseudonyms. Parent-child relationships are encoded into the domain table. An additional table implements the audit trail. An entity-relationship diagram is provided in Figure 3.

ACE features a modular design, with 2 major components in addition to the database: an authentication service, which can also be provided by the operating institution; and a RESTful service providing the user-facing application programming interface (API). All interactions with the database are encapsulated in transactions to guarantee the integrity of the data and to enable rolling back all changes in case of errors.

Technically, ACE is written in Java 21 and uses Spring Boot to implement the RESTful service [22]. Persistence is provided by a PostgreSQL [23] database, with HikariCP [24] being used for connection pooling. Java Object Oriented Querying is used for secure and efficient Structured Query Language query construction and execution [25]. Authentication and authorization are implemented using Keycloak. Every REST end point and domain has its own access right, so that users can be individually granted or denied access to any desired combination of actions supported by ACE and domains. The service can be deployed via Docker.

### Pseudonymization Algorithms

Currently, ACE includes 9 different pseudonymization algorithms. First, we included a variety of commonly used hashing algorithms (MD5, SHA1, SHA2, SHA3, BLAKE3, and xxHash), resulting in outputs between 16 and 128 characters in hexadecimal encoding (between 64 and 512 bits). Moreover, pseudonyms can be generated as consecutive numbers (use with caution, as this may leak temporal patterns). ACE can add checksums (using the Luhn [26] mod N algorithm) to the generated pseudonyms and can pad or cut them to a predefined length. All hashing algorithms use a randomly generated salt.

In addition to providing consecutive numbers, random numbers as well as random characters can be generated of a desired length, with checksums and padding. Generally, the generated random pseudonyms can be represented in different alphabets including hexadecimal, 0-9, A-Z, A-Z0-9, and A-Z0-9 without the letters B, I, O, and S due to their similarity to numbers. This helps to control the lengths of the pseudonyms generated as well as the adaptability to individual needs. For example, hashes can be created for use in automated processing pipelines, and short human-readable representations with check digits for manual use and printing.

When using one of the random character algorithms, the pseudonymization service checks whether the generated pseudonym already exists and performs up to *m* retries, with *m* being configurable by the user. If no new pseudonym can be generated within the available number of retries, an error is returned. To prevent domains from running out of pseudonyms within the configured pseudonym space, they can automatically be configured to produce pseudonyms of sufficient length. For this, the user needs to specify the maximum number of pseudonyms needed (*q*) and a minimum probability of success for filling the complete domain (*T*).

There is integrated logic to ensure that those specifications are met. The probability of generating a new pseudonym in a domain that can hold *k* pseudonyms depends on the number of pseudonyms that has already been generated. With *n* being the number of previously generated unique pseudonyms, we get:



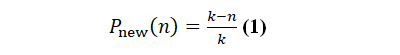



The probability of not drawing a new pseudonym in a single attempt is hence 1–*P*_new_(*n*)=1–((*k*–*n*)/*k*)=*n*/*k*. Therefore, the probability of successfully drawing a new pseudonym in any of the *m* attempts is *P*_attempts_(*n*)=1–(*n*/*k*)*^m^*. The probability that we always generate a new pseudonym when filling the complete domain is hence:



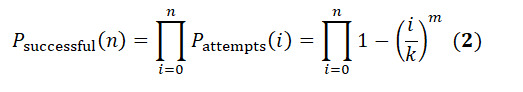



We want this probability to be greater than the minimum success probability *T*, which results in:



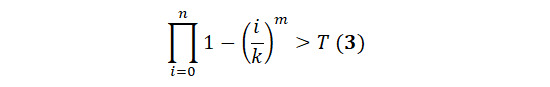



For given parameters *T*, *q*, and *m*, we are interested in *k*, so that *q* pseudonyms can be generated successfully. This can be solved numerically. For *P*_successful_(*n*) with *n*=*q*, conceptually, we try all values for *k*≥*q* until the condition *P*_successful_(*n*)>*T* is met. From the resulting *k*, we can then derive the pseudonym length *l*, given a certain alphabet with size *a*, using 
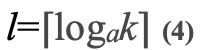
.

As a probability threshold, we can use, for example, the probability to not die from a lightning strike (39 cases between 2000 and 2022 or approximately 2 cases per year in Germany [27]). This corresponds to a success probability of *T*=99.999998%. Table 1 shows how the required pseudonym lengths change for alphabets containing 10 characters and 36 characters, respectively. For a visual representation, see Multimedia Appendix 1.

**Table 1:** Relationship of the pseudonym length *l* in relation to alphabet size *a*, number of retries *m*, and maximum number of pseudonyms *q* for a success probability of *T*=0.99999998.

Maximum number of pseudonyms *q*	Alphabet size (*a*=10)			Alphabet size (*a*=36)		
	Retries (*m*=3)	Retries (*m*=5)	Retries (*m*=10)	Retries (*m*=3)	Retries (*m*=5)	Retries (*m*=10)
10	4	3	2	3	2	2
100	6	4	3	4	3	2
1000	7	5	4	5	4	3
10,000	8	7	6	5	4	4
100,000	10	8	7	6	5	4
1,000,000	11	9	8	7	6	5
10,000,000	12	10	9	8	7	6
100,000,000	14	11	10	9	8	7
1,000,000,000	15	13	11	10	8	7

As can be seen in Table 1, as an example, for a 10-character alphabet with 3 repetition attempts and a minimum success probability of 99.999998%, a pseudonym space with 15 digits is sufficient to store pseudonyms for all inhabitants of Berlin (3.87 million, 12 digits), Germany (84.5 million, 14 digits), or Europe (446.8 million, 15 digits). Using a 36-character alphabet reduces the required pseudonym length to 10 characters.

### Audit Trail and Logging

ACE logs all read, write, update, and delete accesses to the database and provides a full audit trail in a dedicated database table. Which type of event (create, read, update, and delete) is to be recorded for which type of user (ie, which user group, eg, human or technical) can be configured individually for each supported REST operation. This can, for example, be used to design an end point that is only audited for human users or only for nonhuman access by other services. Database access and the creation of audit events are combined into the same database transaction to ensure consistency, for example, in the case of a rollback. The retention period for audit and logging data collected using ACE may differ depending on the use cases and their applicable legal basis. The General Data Protection Regulation, for example, does not specify exact retention periods but states that personal data should be kept no longer than necessary. To accommodate these varying requirements, we implemented a configurable retention period that must be individually set when deploying an instance of ACE.

### Performance Evaluation

We systematically evaluated the performance of ACE by creating a benchmark driver that can emit different types of mixed workloads with different proportions of read, write, update, and delete operations. ACE is accessed by the benchmarking driver in the same way that any other service or user would, including authentication, auditing, and network latency. The benchmark driver is designed to issue as many requests as possible, hence stress-testing ACE for maximum throughput. We benchmarked the singular entity (nonbatch) end points, since these produce the highest overhead when processing requests and recorded the transactions per second (TPS), where a transaction is defined as a successful request.

We studied 3 different types of workloads. The scenario “mostly read” focuses primarily on the operation of resolving a pseudonym (75% of all requests that will be sent are reads) while assuming that around a quarter (23%) of the operations create new pseudonyms. Analogously, the scenario “mostly write” focuses on creating new pseudonyms (75% creates), with resolving pseudonyms being a second frequent operation (23% reads). The “read write” scenario contains a balanced number of creations and resolutions of pseudonyms (49% creates and 49% reads). For all 3 scenarios, from time to time, pseudonyms are updated or deleted (1% each). As a baseline, we also created a “Ping” scenario where we sent 100% of the requests against an end point that responds directly with an HTTP 200-OK status code and performs no database interactions.

To include the impact of the required network connection, we performed all evaluations over the network (10 GbE) on 2 identical Dell EMC PowerEdge R7525 servers, each having 2 AMD EPYC 7502 32 core processors and 512 GB DDR4 RAM. Both servers use Rocky Linux 8.5 as their operating system. All software components (eg, Keycloak and PostgreSQL) as well as the operating systems on the servers were used with their default configuration settings.

On one server, we ran ACE and Keycloak, and on the other server, we ran the benchmark driver for a total duration of 1 hour for each workload scenario. The selected test duration of 1 hour is sufficient to demonstrate long-term stability, as constant performance without degradation could clearly be observed during this period, and the number of generated pseudonyms already exceeded typical case numbers of large hospitals (see Results section).

All requests were made using HTTPS, ensuring that Transport Layer Security encryption was applied throughout the benchmark. The benchmark driver used 128 parallel threads on its machine. ACE was also free to use all available resources. Whenever the Keycloak token’s lifetime (5 minutes) was reached, a token-refresh was issued before the next request was performed.

All requests were performed on a single domain with ACE’s default settings, meaning that the random algorithm was used with an alphabet consisting of letters, resulting in an alphabet size *a* of 26. The length *l* was calculated as 10 using the formula introduced earlier, with *m*=3 retries, *T*=99.999998%, and *q*=100,000,000. Check digits were also calculated and appended.

### Ethical Considerations

This paper covers the design and implementation of a generic research service, which requires no ethics committee approval according to local policies.

## Results

### ACE Application Programming Interface

To its users, ACE offers a REST API following common design recommendations [28,29]. ACE’s end points consider a request header that can be used to set the desired response type (currently either JSON [default] or plain text).

Users can be granted individual rights for each end point, and each supported HTTP method on each domain allowing a very fine-grained management of rights along the cross-product (user×end point×domain×HTTP method). User rights are modeled as subgroups and stored as paths in Keycloak. For example, if the user “foo” has the right to read pseudonyms in the domain “bar” (ie, HTTP method GET on end point “/domain/bar/pseudonym”), the user “foo” needs to be in the group “record-read > bar,” which is stored as “/record-read/bar” in the user’s Keycloak object.

For some end points, we additionally distinguish between standard and privileged versions, providing different functionalities to enable an even finer-grained control over which users and groups can execute those operations. For example, the standard end point for updating pseudonyms only supports updating the validity period, while the privileged version also supports changing the pseudonym itself. Table 2 provides an overview of the implemented end points.

**Table 2:** List of Advanced Confidentiality Engine’s end points and their functionality.

End point	HTTP methods	Notes
/domain	POST, GET, PUT, DELETE	Standard methods for creating, reading, updating, and deleting domains and their configuration.
/domain/privileged	POST, PUT	Methods for creating and updating domains with elevated privileges. This enables access to more configuration parameters, such as the salt, the pseudonym length, and the padding character.
/domains/{domain}^a^/{attribute}	GET	Read access to a specific attribute of a domain.
/domains/{domain}/salt	PUT	Allows overwriting the salt value of an empty domain.
/domains/{domain}/pseudonym	POST, GET, PUT, DELETE	Methods for creating, reading (ie, resolving), updating, and deleting pseudonyms.
/domains/{domain}/pseudonym/privileged	PUT	Method for updating pseudonyms with rights to change all properties.
/domains/{domain}/pseudonyms	POST, GET, PUT, DELETE	Method for batch processing of a given set of pseudonyms in 1 operation.
/domains/linked-pseudonyms	GET	End point to retrieve pseudonyms that are in a different domain but belong to the same identifier.

^a^Curly braces (ie, { and }) denote path variables.

Besides end points for accessing individual pseudonyms, ACE also provides end points for batch processing. This enables reducing network latency and transaction overhead with larger workloads. Moreover, ACE offers an end point that can be used to link pseudonyms across different domains. Here, users can provide a source pseudonym as well as a source and a target domain. ACE will then walk through the domain hierarchy and return all pseudonyms in the target domain that are linked with the given source pseudonym. If the source and the target domains do not have a common parent domain, ACE will return a not-found status.

### Performance Evaluation

ACE achieved a consistent average performance of 5969 (SD 113.4) TPS over all scenarios that did not degrade or change over time, when, for example, the number of stored pseudonyms increased. This corresponds to around 21.5 million transactions in 1 hour. We also systematically evaluated the impact of authentication and authorization. In the “mostly read,” “read write,” and “mostly write” scenarios, the average throughput decreased from 6343 to 6048 TPS (–4.6%), from 6324 to 5977 TPS (–5.5%), and from 6303 to 5882 TPS (–6.7%), respectively. For a visual representation, see Multimedia Appendix 2.

For large-scale integrations, for example, into data lakes, this overhead is not considered a significant bottleneck. First, ACE’s high throughput of about 6000 TPS is unlikely to be the rate-limiting step in any complex data pipeline. Second, our benchmarks were performed on nonbatch end points to measure the maximum overhead. In real-world data flows, ACE’s batch processing capabilities would be used, amortizing the 1-time cost of authentication across many records and making the per-record overhead negligible.

Throughput on the ping end point was 25,200 TPS, meaning that workloads with mostly reads could be performed at about 24%, mixed workloads at about 23.7%, and write mostly workloads at about 23.3% of the network-bound theoretical maximum throughput.

**Figure 4:**
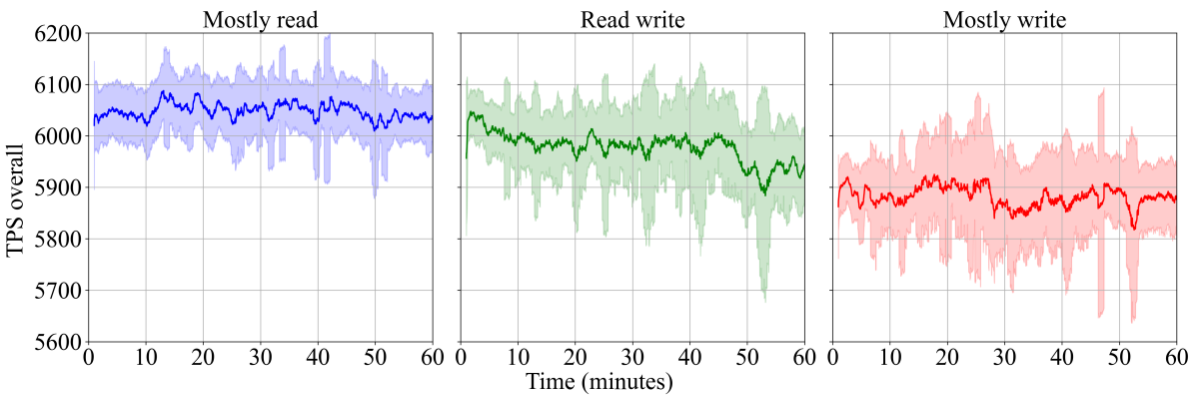
TPS achieved in the 3 scenarios over a 1-hour experiment. Plotted using a sliding window of 60 seconds. TPS: transactions per second.

Figure 4 shows the specific throughput measured over time for the 3 scenarios. As can be seen, the performance is stable and comparable in all settings, with an average of 6048 (SD 69.9), 5882 (SD 100.8), and 5977 (SD 98.2) TPS for the “mostly read” scenario, the “mostly write” scenario, and the “read write” scenario, respectively. Since all database accesses for each request are bundled into a single transaction that also writes to the audit trail, there is no significant performance increase in read-heavy workloads.

Table 3 shows the storage space used by ACE for storing pseudonyms and the audit trail. For a visual representation, see Multimedia Appendix 3. As can be seen, space consumption grows linearly over time, while the amount of space required for the audit trail is about the same over all scenarios. As additional space is needed for the pseudonyms, the slope of the required overall volume increases with the fraction of write operations. As shown in Table 3 (in the 2 rightmost columns), the “mostly write” scenario requires the most space, with 6.56 GiB used for 15.67 million pseudonym records and 3.73 GiB used for 21.17 million audit trail entries.

**Table 3:** Space consumption (in gibibytes) of the Advanced Confidentiality Engine for storing the pseudonyms and the audit trail entries across the different workload scenarios over a 1-hour experiment.

Time (minutes)	Scenario “mostly read”			Scenario “read write”			Scenario “mostly write”	
	Storage for audit event table	Storage for pseudonym table	Storage for audit event table		Storage for pseudonym table	Storage for audit event table		Storage for pseudonym table
5	0.3	0.2	0.3		0.4	0.3		0.5
10	0.6	0.3	0.6		0.7	0.6		1.1
15	0.9	0.5	0.9		1.1	0.9		1.6
20	1.3	0.7	1.3		1.5	1.2		2.2
25	1.6	0.9	1.6		1.8	1.6		2.7
30	1.9	1.0	1.9		2.2	1.9		3.3
35	2.2	1.2	2.2		2.5	2.2		3.8
40	2.5	1.4	2.5		2.9	2.5		4.4
45	2.8	1.5	2.8		3.3	2.8		4.9
50	3.1	1.7	3.1		3.6	3.1		5.5
55	3.4	1.9	3.4		4.0	3.4		6.0
60	3.7	2.0	3.7		4.3	3.7		6.6

## Discussion

### Comparison With Prior Work

One of the first locally deployable pseudonymization services to receive broader adoption is the “PID Generator” implementing the method for generating error-correcting codes suggested by Faldum and Pommerening [30]. As shown in Table 4, the software does not provide advanced features, such as support for domain hierarchies, limiting its flexibility, and lacks an audit trail or validity periods, reducing the ability to control pseudonymization workflows and is not actively developed anymore. However, the implemented methods have been adopted by other software projects. One important example is the open-source software (OSS) “Mainzelliste” by Lablans et al [16]. The service offers a web-based client-server architecture with a scalable REST API for automated access. Mainzelliste focuses on patient registration, record linkage, and the generation of pseudonyms for indexed patients, while our service focuses on the creation of pseudonyms and the management of the connections between pseudonyms and identifiers. Moreover, in contrast to Mainzelliste, our service offers greater flexibility, as it can be used to maintain multiple pseudonyms per patient or study participant and to create chains of pseudonyms, for example, for sharing data with external parties, and offers additional functionalities, such as validity time periods and different pseudonymization algorithms. Bialke et al [15] introduced gPAS in 2015. gPAS is a web-based tool that offers a Simple Object Access Protocol API for automated access (and a REST API if combined with a “Dispatcher” component [31]) and includes a lot of features to customize the generated pseudonyms. gPAS also offers customizable domain hierarchies but no validity periods or attribute inheritance. Introduced in 2017, the Clinical Records Anonymization and Text Extraction tool was developed for pseudonymizing structured data in databases [32]. A major focus of Clinical Records Anonymization and Text Extraction is on free text by incorporating external natural language processing tools, and pseudonyms are created using cryptographic methods. Hence, flexible configurations, for example, through pseudonym domains or validity periods, are not supported.

**Table 4:** Comparison of pseudonymization tools and selected basic and advanced functionalities.

Tool or service	Release year	OSS^a^	API^b^	Basic functionalities	Selected advanced functionalities
PID^c^ Generator [30]	2005	✗	—^d^	Pseudonym generation	—
Mainzelliste [16]	2015	✓	REST^e^	Registration, record linkage	—
gPAS^f^ [15]	2015	✓	SOAP^g^	Pseudonym generation	Domain hierarchies, bulk processing
OpenPseudonymiser [33]	2011	✗	—	Pseudonym generation, record linkage	Bulk processing
CRATE^h^ [32]	2017	✓	—	Pseudonym generation, record linkage	Bulk processing
EUPID^i^ [34]	2014	✗	REST	Pseudonym generation, record linkage	Bulk processing
OPT^j^ [18]	2024	✓	—	Pseudonym generation, registration	Bulk processing
SPIDER^k^ [17]	2022	✗	REST	Pseudonym generation, record linkage	Bulk processing
ACE^l^ (this work)	2025	✓	REST	Pseudonym generation	Attribute inheritance, domain hierarchies, bulk processing

^a^OSS: open-source software.

^b^API: application programming interface.

^c^PID: patient identifier (originally from German Patientenidentifikator).

^d^Not available.

^e^REST: Representational State Transfer.

^f^gPAS: Generic Pseudonym Administration Service.

^g^SOAP: Simple Object Access Protocol.

^h^CRATE: Clinical Records Anonymization and Text Extraction.

^i^EUPID: European Unified Patient Identity Management.

^j^OPT: ORCHESTRA Pseudonymization Tool.

^k^SPIDER: Secure Privacy-Preserving Identity Management in Distributed Environments for Research.

^l^ACE: Advanced Confidentiality Engine.

The space of prior work also includes desktop applications. For example, we recently introduced the OPT, a software designed for rapid deployment in case of health emergencies. However, the OPT focuses to support individual studies that include and register patients or participants and not for high-throughput scenarios [18], and it is not available as a web service. Another example of a desktop application is OpenPseudonymiser [33], introduced in 2011. Its main functionality is to generate hashes for columns in a CSV file. Obviously, these approaches are not suited for large-scale deployments in translational research platforms.

Finally, several software-as-a-service offerings have been established in recent years. One example is the European Unified Patient Identity Management (EUPID) service [34]. The tool is now available at the European Commission—Joint Research Centre as part of the European Rare Disease Registry Infrastructure. EUPID can be accessed through a web application in the European Rare Disease Registry Infrastructure or an API and features record linkage and bulk-processing capabilities. As a web-based service, it has been primarily designed for research networks and requires patient consent and does not support local deployments. Another example is the tool SPIDER [17], which was designed by the European Commission—Joint Research Centre for use in rare disease projects. SPIDER can be accessed through the online SPIDER client or by integration as a plug-in into existing software via an API. The tool features a RESTful API, handles multiple pseudonyms for each identifying data block, and supports linkage. Analogously to EUPID, SPIDER is not available as OSS for local deployments. For a more detailed comparison of pseudonymization tools and services, we refer to a recent review by Abu Attieh et al [14].

In our benchmarks, ACE demonstrated a sustained performance of about 6000 TPS for any of our mixed workloads of creating, reading, updating, and deleting pseudonyms. While the paper introducing the OPT reported a higher number of ~10,000 TPS [18], the underlying benchmark measured a bulk import of 100,000 patient identities, which is a fundamentally different task than the continuous, mixed-transaction scenario we stress-tested ACE with. Moreover, performance evaluations of the tools developed in the project “a modular systematic approach to implement a central data management,” which include gPAS, reported that 100,000 record linkage operations took about 10 minutes (~167 TPS), while 3 million linkage operations took approximately 110 hours (~7.5 TPS) [35]. It is important to note that these numbers cannot be directly compared with each other, as they measure the performance of different operations under different workload configurations and use different hardware setups.

### Limitations and Future Work

ACE has been designed as a scalable service for high-throughput pseudonymization processes as part of translational data processing platforms. It can currently only be accessed via its REST API. As such, ACE cannot easily be used in manual processes, for example, to support individual studies that register patients or participants. Future work will focus on developing a web-based graphical user interface for ACE that can be deployed together with services for patient or participant registration in an integrated manner [20]. This will facilitate its use in a variety of further contexts, and we plan to publish a follow-up report on the experiences gathered.

Moreover, we plan to integrate interfaces following the Health Level 7 Fast Healthcare Interoperability Resources standard [36] based on a recent specification [37]. This API offers 5 core functions including the creation, resolution, and deletion of pseudonyms. A key feature of the API is its support for both single-pseudonym contexts, with 1 pseudonym per individual, and multipseudonym contexts that allow for multiple pseudonyms for the same person. The specification will be used within the national data sharing platform established by the MII in Germany, which also works on interoperability tests for such services. As part of our further ongoing development processes, we also plan to add record linkage [38-40] and patient registration functionalities, which can then directly be integrated with the pseudonymization capabilities to also achieve high performance in such workflows.

We believe that the lack of scalability analyses in other papers on related tools shows that our contribution clearly fills a gap. In future work, we plan to generalize our benchmarking approach and execute it against other services to provide a comparison. This requires quite some work, however, to overcome differences in supported processes and APIs. Finally, the software as well as the operating system’s settings for our experiments were used with their default settings. We believe that by fine-tuning the settings of those components, ACE would be able to scale even further, which we plan to investigate in future work as well.

### Conclusions

We introduced ACE, a modern and scalable pseudonymization service tailored toward the requirements in biomedical research. We presented its architecture and database design as well as the interfaces provided. Using a thorough performance evaluation, we showed that high-throughput pseudonymization services can be offered while still providing advanced features, such as domain hierarchies with inheritance, validity periods, an audit trail, and a wide range of pseudonymization functions. Both ACE and the benchmark driver are available as OSS under a permissive license, together with accompanying documentation [41]. Although designed for the biomedical domain, scalable pseudonymization services, such as ACE, are also important in other domains, for example, when analyzing telecommunications data and customer support logs [42,43].

## Appendix

Multimedia Appendix 1 Relationship of alphabet size *a*, pseudonym length *l*, number of retries *m*, and maximum number of pseudonyms *q* for a success probability of *T*=0.99999998. The left plot shows the minimum pseudonym lengths for an alphabet with 10 characters, the right side for an alphabet with 36 characters.

Multimedia Appendix 2 Impact of the access control (authentication and authorization) on the average transactions per second across the 3 different scenarios.

Multimedia Appendix 3 Space consumption of Advanced Confidentiality Engine for storing the pseudonyms and the audit trail entries across the different workload scenarios over a 1-hour experiment.

## Abbreviations

ACE: Advanced Confidentiality Engine
API: application programming interface
EUPID: European Unified Patient Identity Management
gPAS: Generic Pseudonym Administration Service
MII: Medical Informatics Initiative
OPT: ORCHESTRA Pseudonymization Tool
OSS: open-source software
PROM: patient-reported outcome measure
REST: Representational State Transfer
SPIDER: Secure Privacy-Preserving Identity Management in Distributed Environments for Research
TPS: transactions per second
