# Supplementary figures and images for: The Advanced Confidentiality Engine as a Scalable Tool for the Pseudonymization of Biomedical Data in Translational Settings: Development and Usability Study
Source: J Med Internet Res. 2025 Nov 5;27:e71822. doi: 10.2196/71822 (PMC12631087; doi:10.2196/71822)

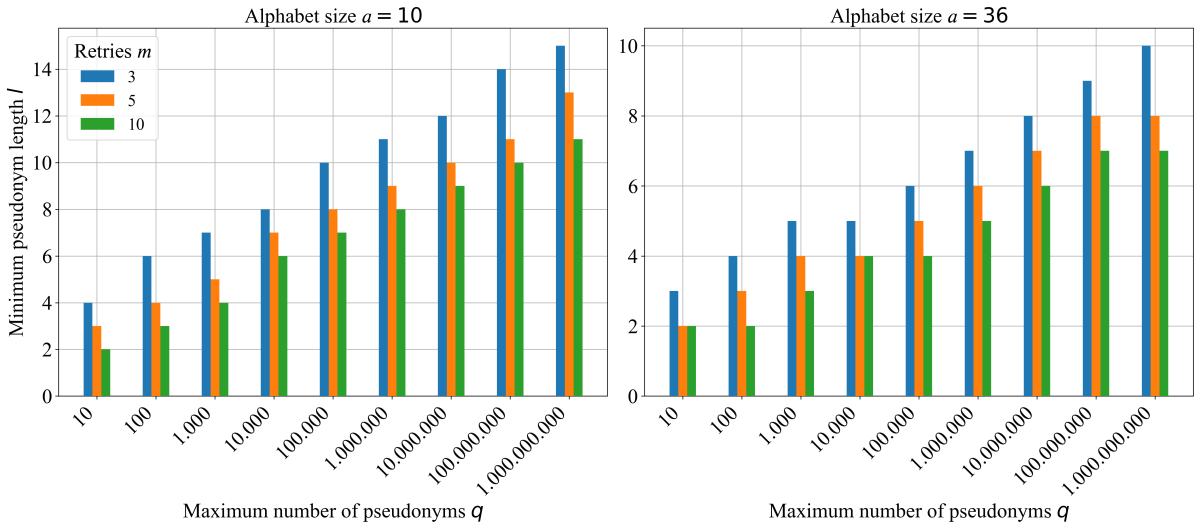

Supplement: Multimedia Appendix 1 [file jmir_v27i1e71822_app1.png]

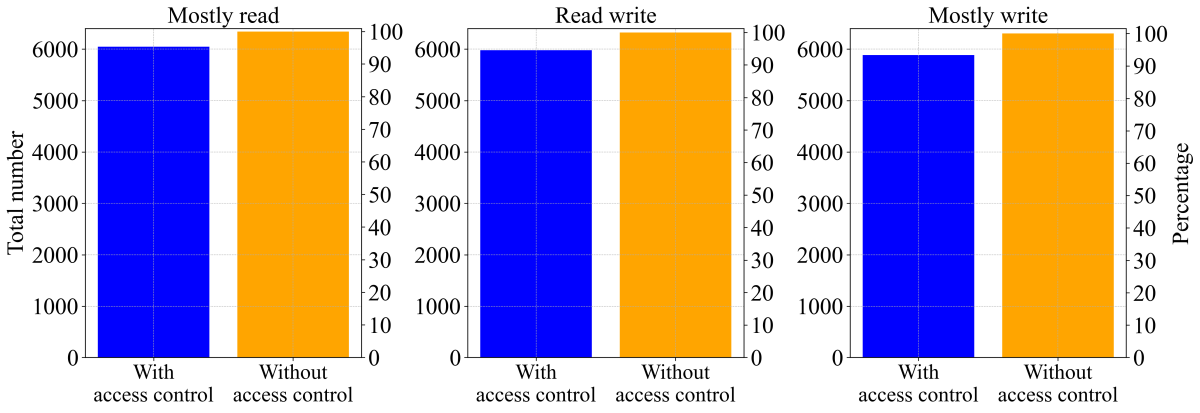

Supplement: Multimedia Appendix 2 [file jmir_v27i1e71822_app2.png]

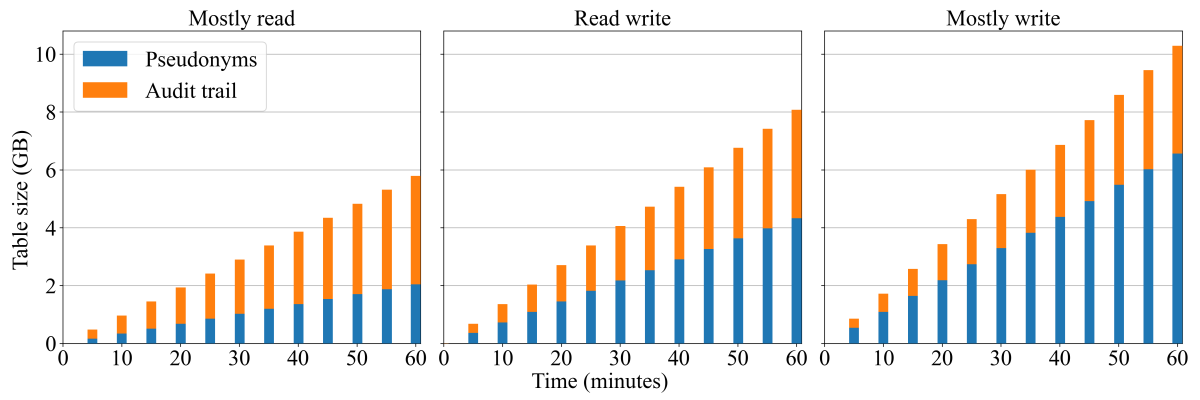

Supplement: Multimedia Appendix 3 [file jmir_v27i1e71822_app3.png]
